# Supplementary material for: Ophthalmic Complications After Dental Procedures: Scoping Review
Source: Diseases. 2025 Aug 4;13(8):244. doi: 10.3390/diseases13080244 (PMC12385263; doi:10.3390/diseases13080244)
Supplement: Supplementary file 1 [file diseases-13-00244-s001.zip › Database Search.pdf]

Database: PubMed

Search Date: September 1, 2024

Search Strategy: (“ocular complications”) AND (dental procedures”)

No filters applied. All languages and publication dates included.
